# Ultrasonic Time-of-Flight Computed Tomography for Investigation of Batch Crystallisation Processes

**DOI:** 10.3390/s21020639

**Published:** 2021-01-18

**Authors:** Panagiotis Koulountzios, Tomasz Rymarczyk, Manuchehr Soleimani

**Affiliations:** 1 Engineering Tomography Laboratory (ETL), Department of Electronic and Electrical Engineering, University of Bath, Bath BA2 7AY, UK; p.koulountzios@bath.ac.uk; 2 Research & Development Centre Netrix S.A., Wojciechowska 31, 20-704 Lublin, Poland; tomasz.rymarczyk@netrix.com.pl

**Keywords:** ultrasound computed tomography (USCT), travel-time, time-of-flight (TOF), transmission tomography, Fréchet kernels, batch crystallisation, calcium carbonate crystallisation, monitoring crystallisation, quality assurance tool

## Abstract

Crystallisation is a crucial step in many industrial processes. Many sensors are being investigated for monitoring such processes to enhance the efficiency of them. Ultrasound techniques have been used for particle sizing characterization of liquid suspensions, in crystallisation process. An ultrasound tomography system with an array of ultrasound sensors can provide spatial information inside the process when compared to single-measurement systems. In this study, the batch crystallisation experiments have been conducted in a lab-scale reactor in calcium carbonate crystallisation. Real-time ultrasound tomographic imaging is done via a contactless ultrasound tomography sensor array. The effect of the injection rate and the stirring speed was considered as two control parameters in these crystallisation functions. Transmission mode ultrasound tomography comprises 32 piezoelectric transducers with central frequency of 40 kHz has been used. The process-based experimental investigation shows the capability of the proposed ultrasound tomography system for crystallisation process monitoring. Information on process dynamics, as well as process malfunction, can be obtained via the ultrasound tomography system.

## 1. Introduction

There is a great need for on-line monitoring in many industrial operations [1,2,3,4]. The state-of-the-art industrial control research is focused on the integration of on-line monitoring and system control theory, which gives great potential to the automation of processes [5,6,7]. Tomographic imaging systems and their recent development have proven to be an alternative and trustworthy method of on-line monitoring offering significant advantages to large-scale processes. Batch crystallisation is a well-established technique in many industrial fields. Crystallisation process involves several control parameters that are important to achieve high quality products. Several sensing techniques have been implemented to gain knowledge of these processes. In the past few years, the ultrasound tomography has been gaining momentum as an imaging modality for a wide spectrum of medical and industrial applications. In this study we investigate the utility of the ultrasound tomography in batch crystallisation.

Ultrasound computed tomography (USCT) has been gaining interest in both medical and industrial applications. Non-destructive testing (NDT) is a field in which ultrasonic solutions are widely used [8,9,10]. USCT also shows promising applications in the energy field and particularly in the oil and gas industry [11,12,13,14,15]. Ultrasonic measurements have proven to offer solutions in fermentation, solidification and crystallisation processes in the food or pharmaceutical industry [16,17,18]. Many recent studies have been focused on tomography’s potential in the monitoring of crystallisation and stirred tanks’ functionality [19,20,21,22,23,24,25,26]. This is the first study that investigates the application of USCT travel-time tomography results for the crystallisation process in stirred tanks.

In this study, we examine the batch crystallisation procedure. Basic industrial crystallisation set-ups consist of the batch, semi-batch and continuous crystallisers [27]. Batch crystallisers are extensively used for the manufacturing of a wide range of high-value fine chemicals. They are generally useful in small-scale operations, especially when working with highly viscous or toxic chemical systems that are difficult to be handled. The yield, purity, morphology, and size distribution of the crystals comprise the quality targets of the process. Changes in the operating conditions such as mixing, temperature profile and solute concentration, determine variations in the growth, nucleation, and agglomeration of crystals. In precipitation systems, mixing at meso- and microscale combined with the additional rate of the reagent are the two key factors that directly control the quality of a reactive crystallisation reaction [28,29]. Subsequently, the control of industrial crystallisers is a major current industrial concern as it is clearly a very important step of process engineering [30] and tomographic imaging is a potential methodology that can offer significant solutions noninvasively via on-line monitoring.

Ultrasonic measurement techniques have been proven efficient to characterise slurry mixtures during crystallisation processes [17,31,32]. State of the art scientific research presents that ultrasound measurements can differentiate between a region containing well-dispersed crystals and a region containing networks of associated crystals. Ultrasonic compression wave absorption and phase velocity spectra data were found to be sensitive to this difference in suspension structure [7]. Thus, both the commencement of the crystallisation and the nature of the final product could be detected by ultrasound. In that sense, USCT has the potential to be the next breakthrough system, since being combined with automatic control can offer automation to large-scale productions.

This work investigates the functionality of ultrasound tomography in industrial crystallisation. Two sections with experimental processes are presented. The first section consists of experimental processes using fine sugar particles and water/sugar suspensions focusing on the ultrasonic imaging in liquid slurry mixture of sucrose/water particles. The second section focuses on reactive crystallisation experiments, such as calcium carbonate crystallisation. The aim of this research is to examine the USCT capabilities in providing useful information on the distribution of phases within flowing mixtures by distinguishing between different suspension particle concentrations or even between differences in compounds’ structural phase.

## 2. USCT Hardware and Imaging Software

### 2.1. Instrumentation Design

**Figure 1:**
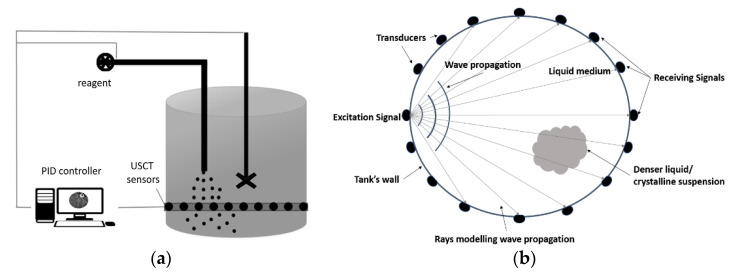
(**a**) Graph depicting ultrasound computed tomography (USCT) applied in an automatic control of batch crystallisation scenario. (**b**) Travel-time tomography functionality (panoramic view).

The proposed USCT system consists of a ring of piezoelectric transducers array, a sensing electronics setup, and a computer system for image reconstruction. These sensors are mounted to the outer surface of the tank using an ultrasonic coupling gel and a belt, keeping the system in place. Figure 1a displays the basic concept of an USCT system for the control of batch crystallisation and generally for stirred tanks environment. The sensor array is connected to the main computer unit, processing signal information and providing tomographic evidence, which leads to the characterisation of the process. The computer unit can also control the parameters involved in the process, namely stirring and injection rate, by utilising a PID (proportional-integral-derivative) controller. The automation of the process can be achieved by aligning the tomographic information to the control of these parameters. This Figure explains the motivation of the current work. Therefore, there is a necessity to distinguish the different phases involved in the crystallisation process. As shown in Figure 1b, the sensors are positioned in a circularly, scanning the cross-sectional plane located 5 cm above the bottom of the tank. The measurement device collects data at the arrival time of the first transmitted pulse allowing travel time imaging. The tomographic data collection is carried out in a parallel fashion, where each transducer has its own transmitting/receiving circuit [33]. The ultrasonic transducers use a centre frequency of 40 kHz, with a sound pressure level close to 97 dB (30 cm/10 V rms). The temporal resolution of the system is 4 frame/s, which is sufficient for this process.

**Figure 2:**
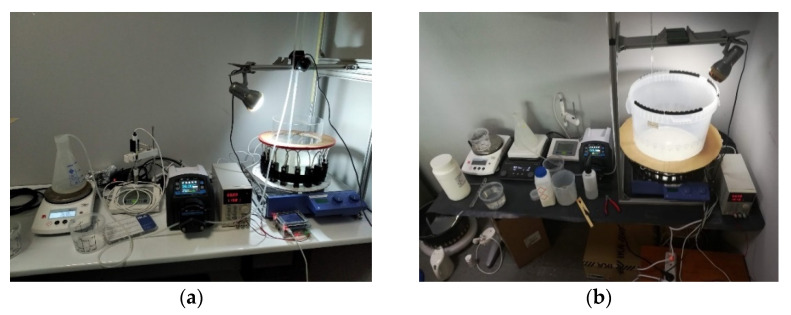
(**a**) USCT ring array integrated to a 20 cm-diameter acrylic tank. (**b**) USCT ring array integrated to a 32 cm-diameter polypropylene tank.

Figure 2a presents the first stirred tank, which is made of acrylic material and Figure 2b shows the second tank, which is made from polypropylene pro-fax plastic, due to its low acoustic impedance. The thickness of both tanks is about 1 cm. The acrylic tank’s diameter is 20 cm while the plastic tank is 32 cm. The sensors are attached to the outer surface, being non-destructive, and the pulses travel through the wall of the tank. As the sensors are attached in the outer surface of the tank’s wall, reconstruction software accounts for the pulses’ penetration of the tank’s wall, to better calculate the time-of-flight (TOF) values providing more accurate raw values describing mainly the medium material’s characteristics.

### 2.2. Simulation Forward Model

A travel-time transmission tomography method was developed and applied to this work. The tomographic system measures the time-of-flight of transmitted pulses. TOF values are calculated by measuring the arrival time of the first transmitted pulse of the captured waveform. The most used approximation for USCT is the ray-based method. It is fundamental for most tomographic schemes. The main advantage is the computational efficiency, since the wave propagation is described using a finite number of rays instead of solving the continuous wave equation. The ray trajectories are calculated by solving the following vector equation and accounting for very high emission frequencies [34].

(1) $$\frac{d}{ds}\left(\frac{1}{c}\frac{dx}{ds}\right)=\frac{1}{c^{2}}\nabla c$$

where s is the arc length along the ray trajectory, x is the position vector, and the *c* is the speed of sound. This, together with the source position and the initial take-off angle, fully specifies the ray. In the case of a uniform sound speed model that only depends on the depth, one can use a convenient parameterisation for the rays which is based on the ray-approximation approach.

(2) $$T=\int_{ray}^{}s\left(x\right)\;dl$$

where, the above integral is based on a single ray path, s denotes the slowness domain $s=c^{-1}$ and finally *T* gives the time of flight of the pulse. Assuming that the ray-path is intensive to a small slowness perturbation, the perturbation in travel-time is given by the path integral of the slowness perturbation along the ray.

(3) $$\delta T=\int_{ray}^{}\delta s\left(x\right)\;dl$$

Since travel-time perturbations given by Equation (3) are insensitive to slowness perturbations anywhere off the geometric ray-path, the sensitivity kernel is identically zero everywhere in space except along the ray-path where it is constant. The implication for ray-based travel-time tomography is that travel-time perturbations should be back-projected purely along the ray-path. A tomographic approach consists of many of these rays, whose amount depends on the angle of the emission beam. All these equations of rays form a system of linear equations, the so-called forward problem. The measurement data formed from subtraction between reference and inclusion data for sound speed led to the reconstructed image of travel-time delays. For a generalised tomographic problem, the above Equation (3) can be expressed as:

(4) δΤ=A δS

where δS is the slowness perturbation, *A* is the modelling operator which describes the sensitivity distribution and δT is the travel-time distribution.

**Figure 3:**
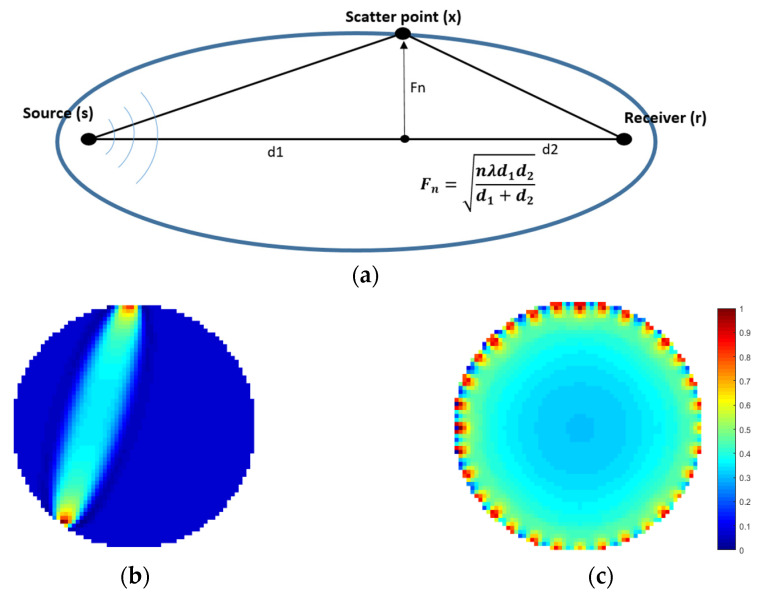
(**a**) Fresnel zone geometry including the calculation of the radius. (**b**) Fréchet sensitivity kernel with a centre frequency of 40 kHz for one measurement. (**c**) Overall sensitivity matrix plot (sum of all sensitivities), displaying the sensitivity distribution of the modelled matrix.

Fréchet sensitivity kernels are based on the first Fresnel zone [35]. Fresnel volume or ‘fat ray’ tomography is an appealing compromise between the efficient ray theory tomography and the computationally intensive full waveform tomography [36]. Using a finite frequency approximation to the wave equation leads to a sensitivity kernel where the sensitivity of the travel time delay also appears in a zone around the fastest ray path. This model assumes that the wave propagation between the source and the receiver has finite high frequency. The travel time is sensitive to an area around the ray-path, typically defined using the first Fresnel zone. The smaller the frequency, the ‘fatter’ the ray kernel. A weighting function based on the travel-time delay dictates that more sensitivity is attributed to pixels nearer the axis of the Fresnel volume, which is the infinite frequency ray path. The sensitivity then decreases linearly from the axis to zero at the edge of the Fresnel volume. For a regular model grid, the Fresnel zone is defined as the region containing all pixels with sensitivity computed based on the transmitter to receiver distance, the signal frequency, and the background velocity [36]. Figure 3 shows the sensitivity maps in our 32-channel system.

The first Fresnel zone is that part of the refracting medium through which energy is transmitted from the source to the receiver within less than a quarter of a period of the ray arrival. In this approach, a weighting function based on the travel-time delay dictates that more sensitivity is attributed to pixels nearer the axis of the Fresnel volume, which is the infinite frequency ray path. To find the first Fresnel zone, we calculate the delay time, Δt, between the first arrival and the arrival of waves that have been scattered at *x*. The delay time is given in Equation (5).

(5) $$\Delta t\left(x\right)=t\left(s,x\right)+t\left(x,r\right)-t_{0}\left(s,r\right)$$

Here t(s,x) and t(x,r) are the travel time from the source (*s*) to x and from *x* to the receiver (*r*) and $t_{0}\left(s,\;r\right)$ is the travel time along the ray path from source to receiver. One can evaluate the times of traveling using ray tracing method. A point *x* is always within the first Fresnel zone if the corresponding travel-time satisfies the following equation, in which T defines the emitted wave’s period:

(6) $$\left|\Delta t\left(x\right)\right|<\frac{T}{4}$$

The following function defines the sensitivity of a Fréchet kernel based on the first Fresnel zone:

(7) $$S\left(x\right)=K\;V\left(s,x\right)\;V\left(x,r\right)\;\cos\left(2\pi \frac{\Delta t\left(x\right)}{Τ}\right)\;exp\left(-{\left(\frac{a\Delta t\left(x\right)}{\frac{T}{4}}\right)}^{2}\right)$$

In this case *S(**x**)* is the sensitivity at ***x***, V(s,x) is the amplitude of the wave field at point ***x*** propagating from point ***s*** (source), V(x,r) is the amplitude at point r propagating from point ***x*** (scattered wave) and *K* the normalisation factor. The sensitivity formula has been developed on the Fresnel zones method [37,38]. SIPPI MATLAB software (Hansen, Copenhagen, Denmark) has been used to generate these sensitivity kernels [39]. In this work we used Fréchet sensitivity maps described by Buursink et al. [36].

### 2.3. Image Reconstruction

The internal time delays are reconstructed by using pulse ΔT from a change in the measured boundary slowness ΔS. A linear forward operator can be described by

(8) ΔT= A ΔS+e

where A is the forward operator and e is the noise in the measurements. ΔS is defined as the acoustic slowness profile of the scanned region in different discrete times and ΔT as the sensors’ TOF measurements. A simplified inversion can be done using back projection method such as:

(9) $$\Delta S\;\approx \;A^{T}\;\Delta T$$

Total variation regularisation (TV) [40,41,42] has a greater potential in solving regularised inverse problem in a stabilised fashion. The TV problem is defined as an optimisation problem:

(10) $$\min A\left(\Delta S\right)={\left|\left|\left(A\;\Delta S+e\right)-\Delta T\right|\right|}^{2}+\;a\;{\left|\left|\nabla \Delta T\right|\right|}_{1}$$

where a, the regularisation parameter, ∇ is the gradient and ${\left|\left|.\right|\right|}_{1}$ is the $l_{1}$−norm. Then the problem to be solved is the constrained optimisation problem as shown in Equation (12).

(11) $$x_{a}=\arg\min{}_{\Delta S}\left(\;\alpha \;{\left|\left|\nabla \Delta S\right|\right|}_{1}\right)\;\mathrm{such}\;\mathrm{that}\;\;{\left|\left|A\;\Delta S-\Delta T\right|\right|}^{2}<p$$

This is solved by the split Bregman-based TV algorithm [40]. After a few manual parameter adjustments, we chose global values of regularisation parameters that provide optimal imaging results. The algorithm offered a good response by deleting the undesired artefacts.

To analyse the experiments related to crystalline particle imaging and crystallisation we use some quantitative information from data and the image. We calculate the average values of TOF delays, namely of the subtraction of background from full data, for every recorded frame.

(12) $$x_{k}=\frac{1}{M}\overset{M}{\underset{i=1}{\sum }}{\mathrm{Full}}_{n,m}-{\mathrm{Back}}_{n,m}\;,\;k=\left[1,\;\ldots ,\;K\right],\;n=\left[1,\ldots ,N\right],\;m=\left[1,\ldots ,M\right]$$

where *M* is the 256 receiving data coming from the tomographic device and *N* is the number of captured frames.

## 3. Crystalline Particle Imaging

This section presents experiments for USCT on the combination of fluid and solid particles. Specifically, its main purpose is to analyse the TOF responses in inhomogeneous two-phase media. In the first two experiments fine sugar particles were used as the adding substance to the liquid medium, without any stirring being applied. In the third experiment a water/sugar suspension was injected, while stirring has been applied using a magnetic stirrer. In all three experiments, a temporal resolution of 10 fps was applied (without accounting for reconstruction time for fps). An acrylic circular tank of 20 cm diameter, filled with tap water, and a ring of 16 ultrasonic transducers were used in all experimental set-ups of this section.

### 3.1. Continuous Sucrose Particles Pouring

**Figure 4:**
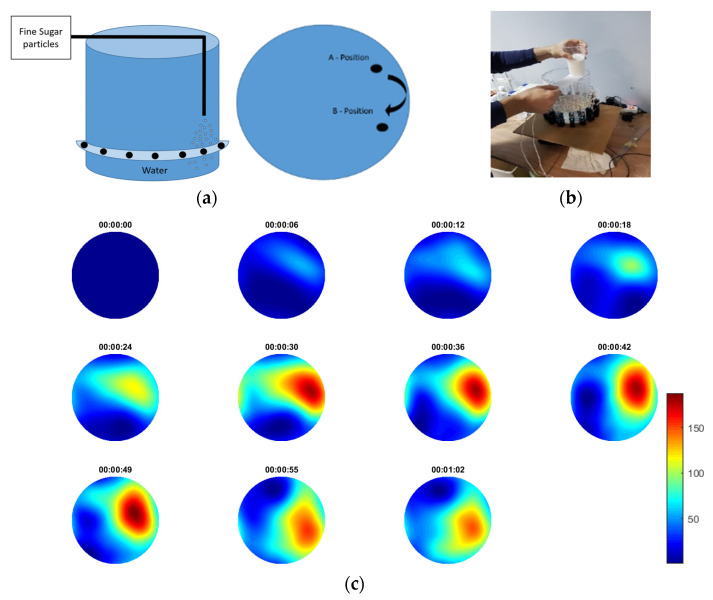
(**a**) Schematic of the experiment with panoramic view of the injection points of the experiment. The transition from A to B occurred 29–44 s from the starting time. (**b**) Photo of the experiment. (**c**) Reconstruction with the specific capture time. Scale bar describes pulses’ travel-time delays.

In the first experiment, fine sugar particles were thrown in the medium, at a steady, continuous pace. Figure 4a shows a schematic of a planar and cross-sectional view of the tank, including the injection positions and the circular ring of ultrasonic transducers. Figure 4b shows a photo of the actual experiments. The recording happened on-line with the process and it started before and finished after the pouring of the solid sugars. Pouring was continuous and lasted for 55 s. The injection started from position A and after 29 s started moving to position B. This movement lasted for 15 s. Then stayed at B until the end. The injection stopped 5 s before the end of capturing frames’ sequence. Figure 4c presents the results in frame data with the recorded time as a reference. The results are being presented in a travel-time mapping thus, the scale presents delays of the first arrived pulses. Note that as the sugar particles poured into the water, some time was needed before they started precipitating to the bottom. Thus, one can notice the existence of lower difference TOF values, at first, then higher and then lower again. This fact was due to the sedimentation of the sugar particles, as no stirring was used. The scale bar of the reconstructions shows TOF delays that goes up to 155 ms, which are considered high values and occurred due to the abrupt changes in homogeneity of the medium, due to the continuous high pouring rate.

### 3.2. Noncontinuous Sucrose Particles Pouring Using a Mechanical Rotator

**Figure 5:**
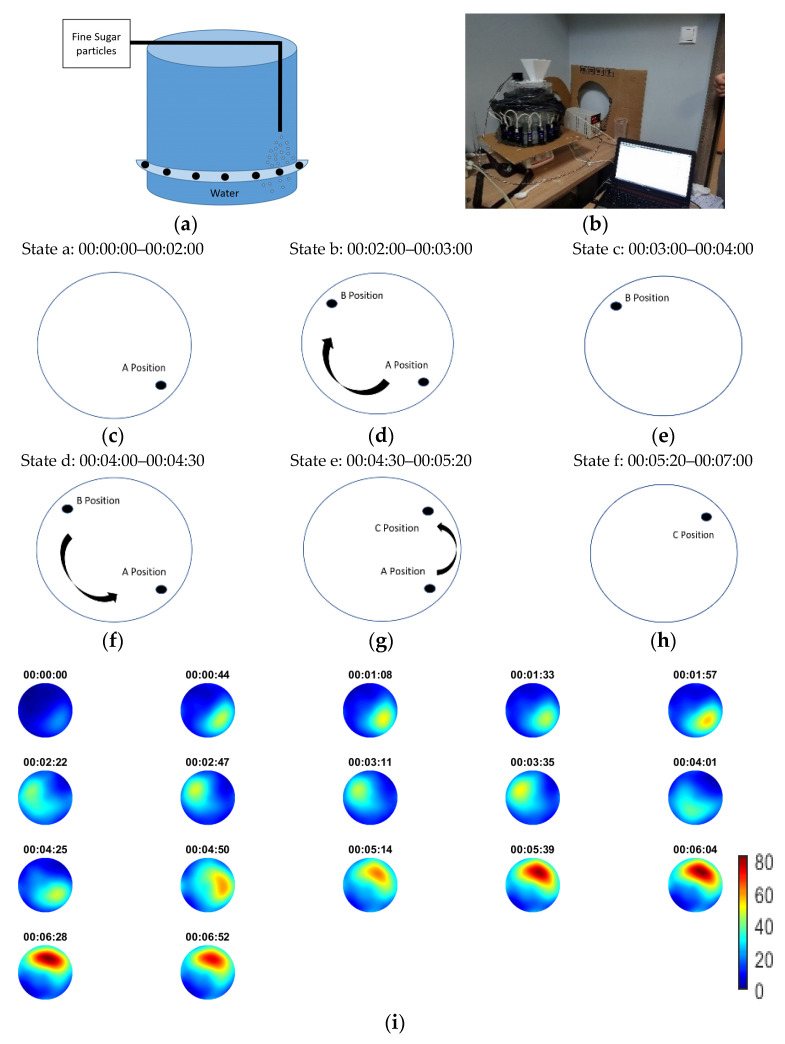
(**a**) Schematic of the tank. (**b**) Experimental apparatus photo. (**c**–**h**) Graph explaining the shifting of injection position. (**i**) Reconstruction of time-of-flight (TOF) delays with the specific capture time. Scale bar describes pulses’ travel-time delays of the first pulse.

This experiment aimed at ascertaining the efficiency of the USCT device in real-time measurements and in a continuous change of the injection point. Sugar particles were fed into the medium (contaminated water) as shown in Figure 5a. A mechanism was used to apply noncontinuous pouring. The mechanism rotated the plastic layer that consisted of four holes. The holes let a specific amount of sugar be released into the water each time. Furthermore, the point of injection constantly changed during the process. Figure 5a,b depicts a schematic and a photo of the experimental set-up, respectively. Figure 5c–h presents the six stages of the experimental process with the corresponding time reference, which describes the movement of injection point. The whole process lasted for 7 min. As the sugar particles needed some time to start sedimentation and the layer of the sensor’s ring was a few centimetres below the surface of the water, the first denser regions started becoming obvious a few frames after the beginning, as the reconstructed frames show in Figure 5g.

The reconstructions agree with the injection’s shifting position. The intermittent injection drove a continuous change of particles’ concentration over time, being at a high then lower and then higher level, again. This change of the particles’ concentration within the field of view translated into higher then lower and then higher again delays of TOF signals. After 4:30 min from the start of the process, the injection point passed through position A again, which drove of sugars in the region to higher concentration. Therefore, one can notice a significant increase of TOF delays in the last reconstructed frames. Moreover, the injection stayed a bit longer between position A and C and, subsequently, as the experiment approached the end, bigger TOF delays could be noticed.

### 3.3. Injection of 75% (kg/mol) Sucrose/Water Suspension

**Figure 6:**
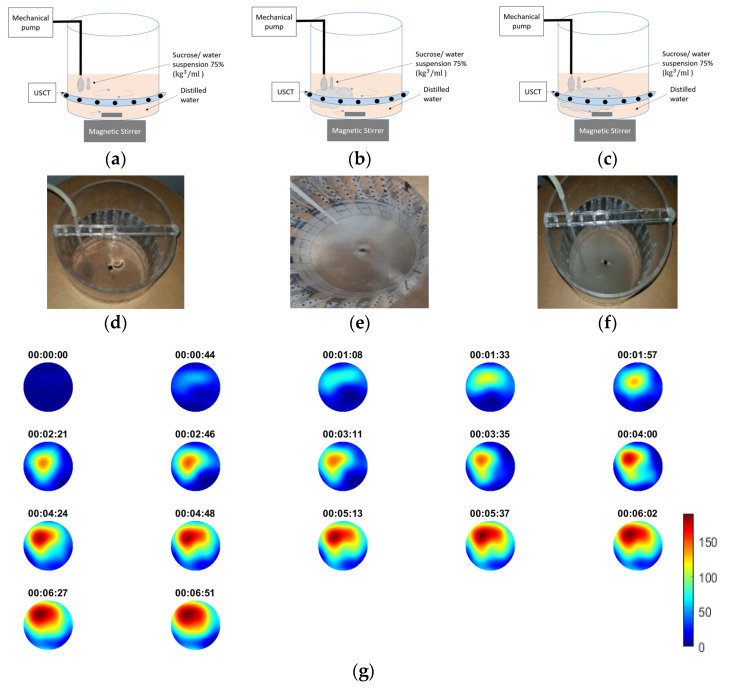
(**a**–**c**) Schematic of the experiment with corresponding photos. (**d**–**f**) Experimental photos of the start, the middle and the end of the process, respectively. (**g**) Reconstruction with the specific capture time. Scale bar describes pulses’ travel-time delays.

In this experiment, a sucrose/water suspension of 75% kg/mL was used as a tracer. Automatic injection by using an electronic pump was applied. The feed rate speed was 35 mL/min. During the process, which lasted for 7 min, mixing occurred. Stirring speed was at 40 rpm. This rate was assumed to be moderate as no swirl effect [43] was depicted. The first higher concentrated regions started forming in the position of injection, driving the first TOF delays. The tank’s dynamics allowed the propagation of the suspension in the medium. As the medium turned more inhomogeneous, so bigger delays of the first arrival pulse could be noticed. Figure 6a–c depicts the experimental process and the different stages of the suspension’s propagation with the corresponding time reference.

Experimental photos and schematics are displayed to clearly present the experimental set-up. Figure 6g depict the reconstructed images over time. High TOF delays up to 155 ms at the end of the process show the high-concentrated regions with the sucrose suspension. Within the frames one can notice the propagation rate as well.

### 3.4. Analysis of Experimental Results

**Figure 7:**
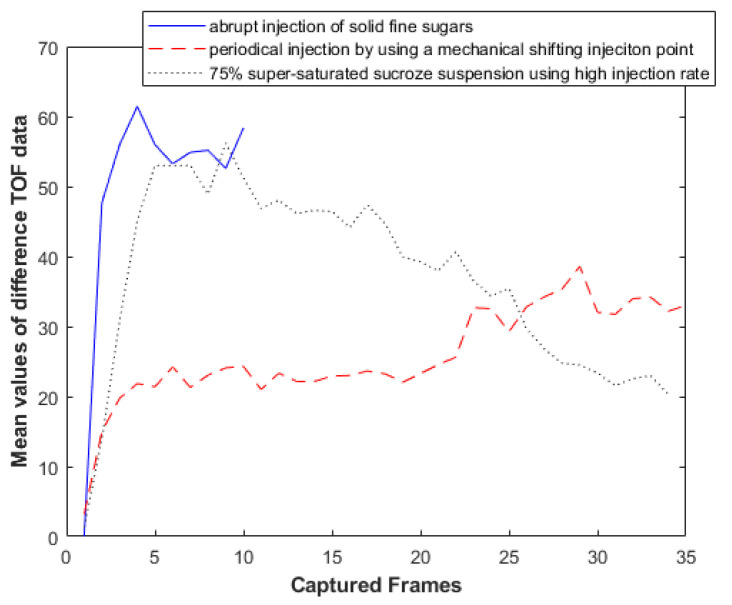
Plot of all frames’ mean value of difference data. The ascending trend of the graph reveals the gradually increasing delay that was introduced to the raw TOF data. The first experiment with the pouring of solid particles of fine sugars lasted for a lot less than the others, according to its nature.

Figure 7 presents a graph of the mean value of all the recorded measured data of the first, second and third experiment, respectively. These values depict travel-time delays over frame. For every recorded frame, there were 256 values from 16 sensors each time. These values were the TOF delays, coming from the subtraction of the background from the full measurement data, using Equation (12). The blue graph presents the first experiment of that section. This function is described by an ascending trend with a significantly high slope, due to the abrupt pouring of the fine sugar. The function became smaller over time, as the corresponding experiment lasted for a shorter period. The red graph, which comes from the second experiment, has an ascending trend as well. The major difference between the two functions is that the slope of the one related to the first experiment is bigger than the one related to the second one. Moreover, the blue graph consists of higher TOF values. These facts come from the different method of pouring. In the first experiment, the pouring rate was abrupt and continuous, while, in the second case, the pouring was periodical. The black graph is related to the experiment of sucrose/water suspension injection. Comparing the black and the red graphs, one can notice the higher values of the black graph, despite that the two experiments lasting for the same amount of time (8 min). According to the use of solid particles in the first experiment, one could expect higher TOF values. The stirring process helped to create a uniform material distribution and reduced the sedimentation effects. This drove the higher delays of the signal as the rate of disturbances were also high. Furthermore, some additional TOF delays were introduced due to the stirring itself.

## 4. Reactive Crystallisation Imaging

**Figure 8:**
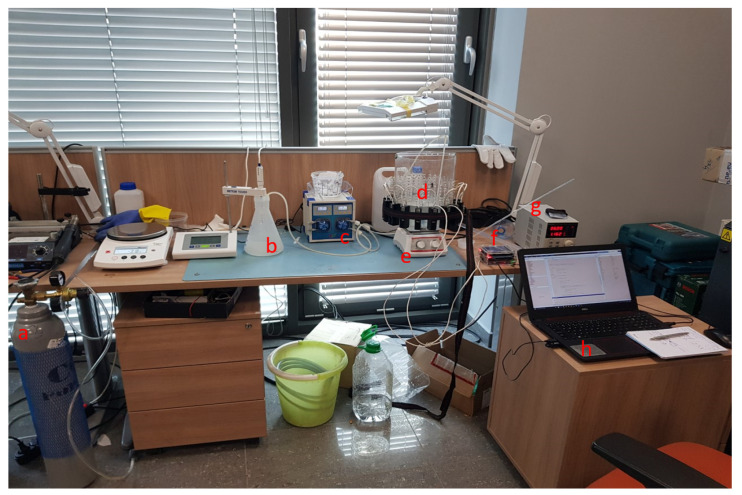
Photo of the batch crystallisation experimental apparatus. (**a**) Carbon dioxide tube for gas injection; (**b**) reagent vessel filled with sodium carbonate solution (reagent); (**c**) electronic peristaltic pump; (**d**) receiving tank filled with calcium chloride solution; (**e**) IKA Midi 1 digital magnetic stirrer; (**f**) tomographic device developed by NETRIX company; (**g**) power supply; (**h**) computer unit used for running MATLAB code and providing tomographic results.

In this section, lab-scale batch crystallisation experiments were conducted. The experimental apparatuses were designed to be aligned to industrial framework. The current study focuses on stirred crystallisation tanks [44]. Calcium carbonate reactive crystallisation was performed [45]. Non-stirring experiments were previously conducted to test the response of the system in the actual crystal formation. Full scale crystallisation experiments (using stirring) were conducted at the end. In a batch concept, the stirring process is necessary, although the non-stirring experiments can give important indications of TOF responses on crystalline slurries without accounting for tank dynamics. Figure 8 shows the experimental set-up by naming all the used equipment. A 32 cm propylene and a 20 cm acrylic tank with the integrated ultrasonic tomographic device were used among the experiments. An electronic peristaltic pump and an IKA Midi 1 (ΙΚA, Cologne, Germany) digital magnetic stirrer were fitted to the stirred tank. The basis of the crystallisation process is the reaction of a sodium carbonate solution (reagent) with a calcium chloride solution. The chemical reaction is described in Equation (13).

(13) $$CaCl_{2}^{aq}+Na_{2}CO_{3}^{aq}\to CaCO_{3}+2NaCl^{aq}$$

The first step of the experimental procedure was the preparation of the reagent. Three hundred grams of sodium hydroxide was dissolved in a vessel filled with 1 L of demineralised water. A carbon dioxide dosing tube and pH meter probe were used to control the pH of the sodium hydroxide. The sodium hydroxide solution was gassed with carbon dioxide to reach and maintain a pH of about 11, resulting to an 1 M ${\mathrm{Na}}_{2}{\mathrm{CO}}_{3}$ (sodium carbonate) solution was prepared in the reagent’s vessel. The solution remained stable for 48 h because of the large amount of heat released by the reaction and the presence of gases. The reagent solution was injected into the receiving tank using a peristaltic pump. A silicone tube was introduced into the sodium carbonate reagent and passed through the pump, the other end of which was on the surface of the liquid in the receiving tank. The receiving tank was filled with two litres of 1 M ${\mathrm{CaCl}}_{2}$ (calcium chloride) aqueous solution. The tank with the installed ultrasonic tomograph was placed on the digital stirrer. The crystallisation reaction took place while pumping the aqueous solution of ${\mathrm{Na}}_{2}{\mathrm{CO}}_{3}^{\mathrm{aq}}$ into calcium chloride at a fixed speed. During the experiment, mixing started in the tank with the ${\mathrm{CaCl}}_{2}$ solution.

### 4.1. Nonstirring Crystallisation

**Figure 9:**
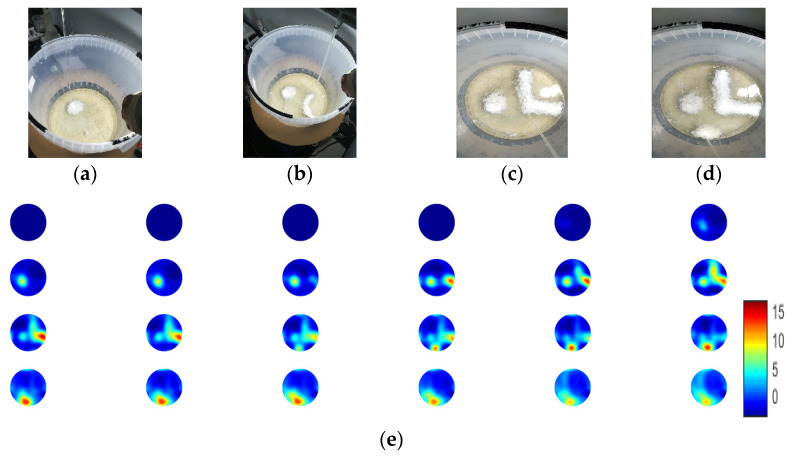
(**a**–**d**) Photos of the experiment and reconstructions. (**e**) Reconstruction with the specific capture time.

**Figure 10:**
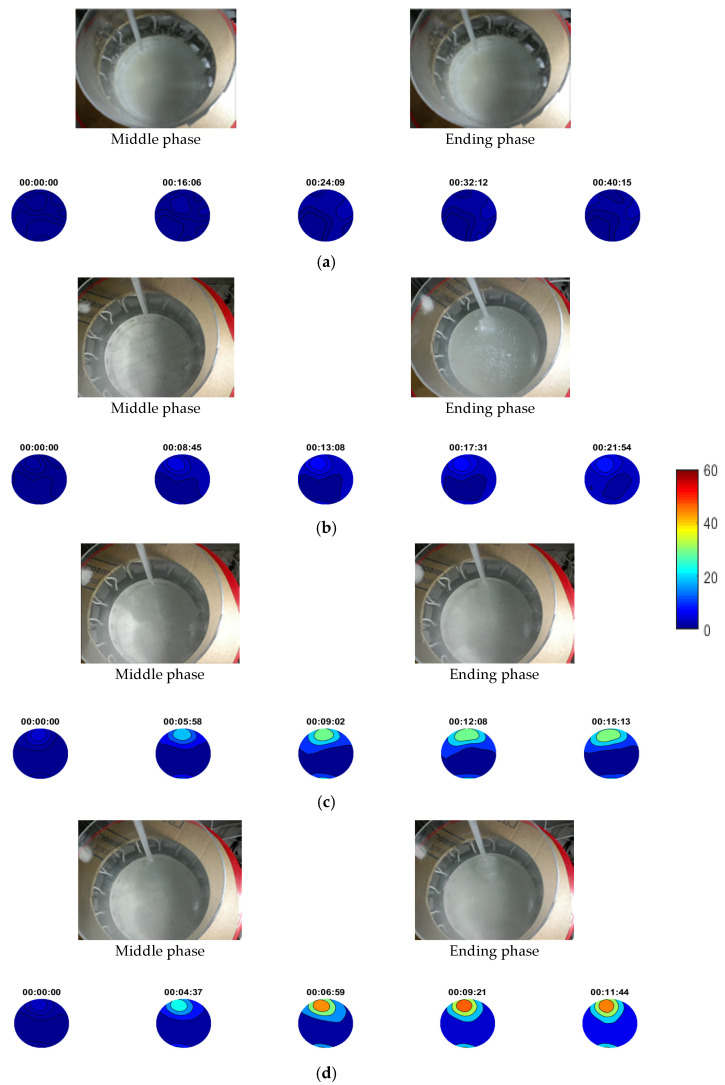
Reconstructions of non-stirring calcium carbonate crystallisation experiments. (**a**) 9 mL/min, (**b**) 18 mL/min, (**c**) 27 mL/min and (**d**) 36 mL/min injection rate. Note that a uniform image scale bar is used for all four sets of experiments to provide comparative evaluation. Scale bar describes pulses’ travel-time delays.

Figure 9 and Figure 10 present the non-stirring calcium carbonate crystallisation experiments. Due to the lack of dynamics, immediate crystalline suspensions were formed at the location where the injection point was. Crystallisation occurred regionally and at a faster pace. In the first non-stirring experiment, which is presented in Figure 9, the injection point was shifted manually. Therefore, the formation of crystalline suspensions followed the movement of injection. For this experiment, the 32 cm propylene tank was used. The reagent’s feed rate was 20 mL/min. Denser suspensions were immediately created, during the injection of the reagent. The denser suspensions started the sedimentation stage, while the suspensions settled down in bottom of tank, they passed through the FOV (field-of-view) of the 2D ring of the sensors. Figure 9a–d shows photos from the experiments in different moments. The reconstructed results agree with the shape of the forming crystals, presented in Figure 9e. An interesting point is that regions with a constant injection over a point reached high TOF delays values, and when the injection point moved, the region went to lower values again. This was due to the immediate crystal formation, especially in this type of reactive crystallisation.

However, when the injection point moved, the crystallisation in the region stopped and subsequently already created crystalline suspensions whether start dissolving or start the sedimentation process. Therefore, one can notice this decay of TOF delays values after the moving of the injection in the reconstructed frames.

Figure 10 presents a sequence of non-stirring calcium carbonate crystallisation using four different injection rates of 9, 18, 27 and 36 mL/min. The injection point was the same in every case. As no stirring was happening, the crystalline suspensions tended to form in a specific location while the medium got denser, as shown in the experimental photos in Figure 10. Photos were taken at two specific points describing the middle and ending experimental phase, in every case. Two litres of calcium chloride solution were used as reagent in all processes. Different injection rates can be translated to a faster experimental process. Recording took place from the beginning, until the end of injection process. Therefore, the number of reconstructions was related to the rate of injection. An averaging method over 100 frames was applied, to eliminate the existed noise coming from stirring conditions.

Reconstructions provide clear depictions of the localised forming suspensions in almost all the cases, as a gradual increase of TOF values over time. In the first case of 9 mL/min, no localised suspension was noticed due to the nature of the low injection rate. Comparing all the cases, the results show a quantitative relation between the travel-time imaging and the concentrations of the forming suspensions, since the increase of the injection rate turns the suspensions more concentrated. A common scale is used for the presentation of results to point out this fact. Contours were applied to clearly distinguish the higher concentrated regions of the tank. The tomographic system proves accuracy by monitoring efficiently almost all the experimental cases and providing a quantitative scale of TOF values related to the concentration of crystalline particle in the forming suspensions.

### 4.2. Stirring Crystallisation

Four experiments with stirring calcium carbonate crystallisation were conducted aligned to industrial standards. Four different reagent injection rates of 9, 18, 27 and 36 mL/min were applied. The injection point was the same in every case. Two litres of calcium chloride solution used as reagent in all the processes. Experiments lasted different amounts of time related to the rate of injection. Recording took place from the beginning until the end of the injection process and an averaging method over 100 frames was applied.

**Figure 11:**
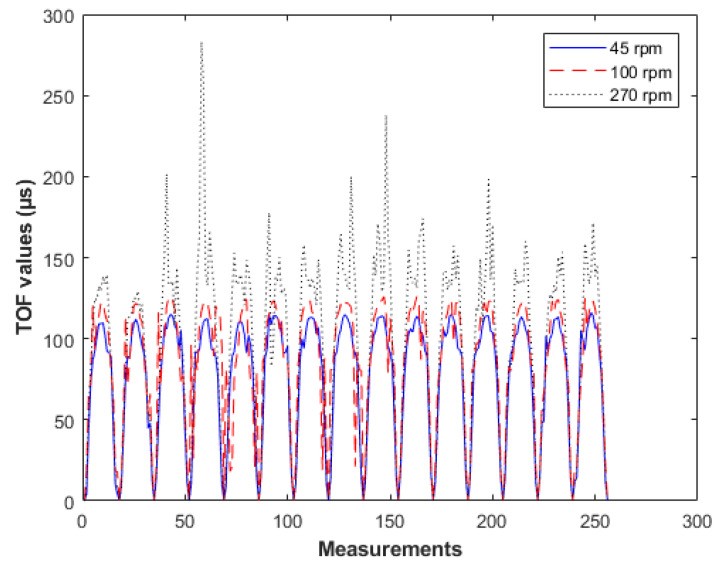
Plots of time-of-flight (TOF) data from background measurements using three different stirring rates of 45, 100, 270 rpm. The tank was filled with calcium chloride.

The stirring process created significant changes in the forming suspensions and the aim of these tests was to inspect and analyse the system’s responses in stirred tanks and crystallisers. A good combination of the mixing factor and the tank’s kinetics led to a uniform distribution of crystalline suspensions across the whole medium. This fact significantly affected the crystal yield. The optimal agitation is the minimum stirring rate needed to keep all crystal particles suspended from the bottom. Therefore, the size distribution and concentration of particles vary within an agitated suspension according to the dynamical state of the medium [46]. Stirring rate can affect seriously the tomographic signals by creating disturbances in the medium. Usually swirls are created which affects the signals and subsequently the reconstructions [22,43,46]. Assessing the stirring rate of the 20 cm tank, a few different rates of stirring were applied while recording. Figure 11 shows recorded data from the tank filled with calcium chloride solution in rates of 45, 100 and 270 rpm. The graphs clearly show the increasing noise factor as we go to higher stirring rates. However, we noticed that when the stirring speed reached 100 rpm, the noise was not significant. Therefore, this rate was picked leading to an eliminated noise effect.

**Figure 12:**
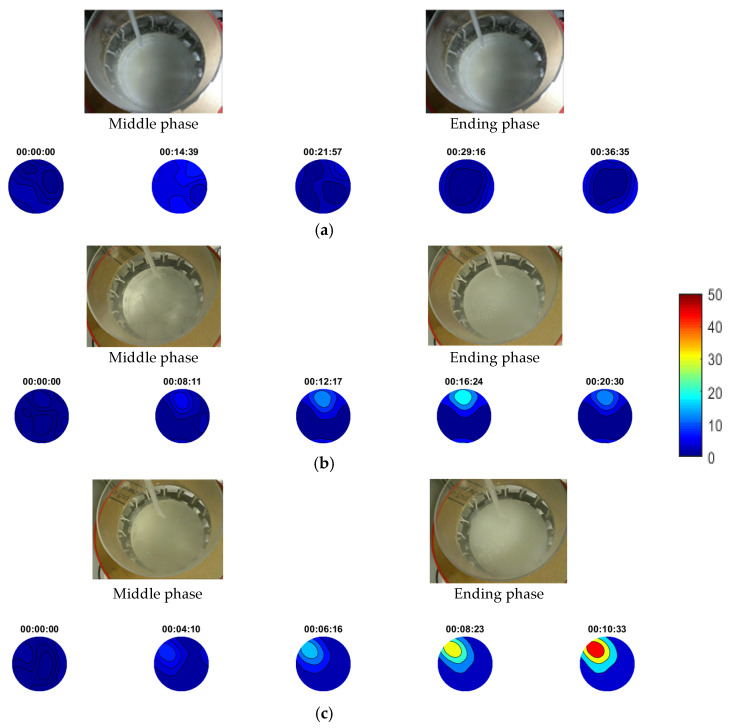

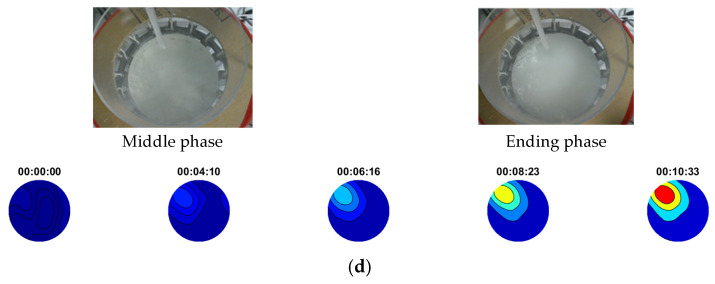
Reconstructions of stirring calcium carbonate crystallisation experiments with (**a**) 9 mL/min, (**b**) 18 mL/min, (**c**) 27 mL/min and (**d**) 36 mL/min injection rate. Stirring rate was at 100 rpm using magnetic stirrer. Note a uniform image scale bar is used for all four sets of experiments to provide comparative evaluation. Scale bar describes pulses’ travel-time delays.

Figure 12 presents experimental photos and the results from stirring reactive crystallisation. Experimental photos describe the middle and ending phase in every case. In all the cases, the tomographic system was capable of reconstructing regions with higher concentrations of crystalline particles, as shown by the contours reconstructions. As regards the reconstructions’ scale, the system showed capability in distinguishing between cases of different injection rates, as higher concentration suspensions tended to be formed by higher injection rates. In the 18, 27 and 36 mL/min cases, the injection rate seemed to be high enough comparing to the tank’s dynamical state since the system detected constant localised forming suspensions. Nevertheless, in the 18 mL/min case, suspensions seemed to start dissolving at some point and thereafter because of the dynamics. In the last two higher-injection-rate cases, stirring could not affect the propagation of suspensions at all. On the other hand, the first case of 9 mL/min seems to consist of a good combination of injection and stirring rate, as reconstructions did not detect localised forming suspensions. Higher TOF values could be noticed in the beginning of the experiment, close to the injection point, but they lowered quickly as stirring turned the medium more homogeneous. This fact led to a better crystal yield. Regarding the three higher injection cases, the more concentrated regions reveal bad conditions of the experimental procedure and subsequently rougher crystals should be expected.

### 4.3. Analysis of Experimental Results

**Figure 13:**
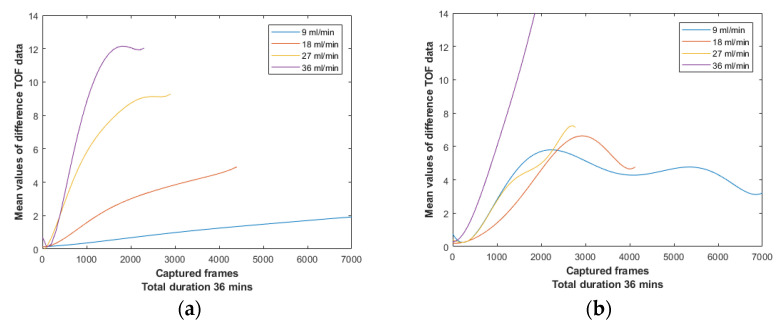
(**a**) Plot of all frame’s mean value of difference data for non-stirring cases. (**b**) Plot of all frame’s mean value of difference data for stirring cases using a IKA Midi 1 digital magnetic stirrer at 100 rpm.

Figure 13a depicts the difference data mean values of the four non-stirring experiments of this section. These graphs were computed by Equation (12). One can expect higher medium inhomogeneities in non-stirring cases rather than in stirring cases, thus, comparing the graphs between Figure 13 one can notice a higher rate of change. Stirring always made the medium more homogeneous avoiding supersaturation. In addition, the most significant conclusion is the ascending trend of the mean value in almost all the cases over time, which assures the efficient detection of higher concentrated mixtures by the device.

A quantitative relationship between the TOF delays and the localised forming suspensions can be noticed in non-stirring cases. The quantitative relationship is an ascending trend of the maximum TOF values while the injection rate increases, as shown in Figure 13a. Figure 13b presents the mean values of difference data from stirring experiments. Because of the amount of noise introduced by stirring and the different means of suspension formation, the system was unable to provide a clear relationship of the TOF delays of these experiments, in comparison to the non-stirring cases. For instance, graphs of 18 and 27 mL/min become really close after the 1500th frame. Moreover, in 9 mL/min case, one can notice a high slope of the graph at the beginning of the experiment, which could be a result of the stirring noise factor. However, after the 1800th frame, it starts to stabilise and then slightly reduces over time, due to the gradual dissolving of the suspensions in the medium.

Examining the stirring experiments, one can realise the importance of the different combinations of stirring and injection rates. In the 9 and 18 mL/min cases, a dissolution of the forming suspensions can be noticed after a point, which caused slightly decreased TOF values. On the other hand, this could not be seen in the two higher injection stirring cases (27, 36 mL/min), since 100 rpm was not enough to dissolve formations of such a high injection rate. Subsequently, an efficient combination of stirring and injection should assure a homogenous formation so that the localised suspensions, or even the direct dissolution of them are not occurring.

These results display a promising potential of travel-time ultrasound tomography not only for monitoring the process by detecting and reconstructing regions with higher crystalline particle concentration but also for quantifying different formations of crystalline suspensions by utilising the scale of the travel-time delays.

## 5. Conclusions and Remarks

This paper presents a travel-time ultrasound tomography for monitoring batch crystallisation processes. The system was first demonstrated in monitoring processes consisting of crystalline suspensions in a nonstationary setting. This followed the monitoring of the calcium carbonate crystallisation reaction. The study examined the combination of injection and stirring factor to the process through the results of the ultrasound tomography system.

The travel-time ultrasound tomographic system provided satisfactory responses in almost all the cases. This has been shown based on a gradual increase of TOF differences due to the gradual crystalline suspension formation over time. The device also shows a good response to distinguishing between different material phases, such as liquid solutions and crystalline liquid suspensions. Experimental results in Section 3 display the distinction between suspensions of various crystalline concentrations.

A calcium carbonate crystallisation reaction aligned with industrial standards is shown in Section 4. The device provided good results in the non-stirring experiments. The experiment of shifting the injection point, showed accuracy in tracking the crystalline formation, following the injective disruption, over time. Analysing the other non-stirring experiments showed a promising potential of travel-time ultrasound tomography not only for monitoring the process by detecting and reconstructing regions with higher crystalline particle concentration but also for quantifying the different forms of crystalline suspensions by utilising the scale of the travel-time delays. The results agreed with the theoretical approach that sound propagates slower in high-concentration suspensions. Our approach showed good potential for distinguishing between material phases and monitoring high-material-phase-contrast processes. As regards the stirring experiments, verifications were applied in the adding component, namely different feed and stirring rates have been applied. Both factors affect significant process outcomes. The stirring factor was found to be extremely important for ultrasonic monitoring since it affects the propagation of the excitation pulses. Through these tests, significant conclusions about the process itself have been made, such as the definition of the adequate stirring rate that this specific process needs to avoid aeration of the liquid compounds, and the optimal combination of stirring and feeding.

For low-feed rates, there is a more uniform image showing that the process of crystallisation occurred without any localised issues. With higher rate of injection, the USCT device showed the localised changes in TOF images, which could lead to localised crystallisation. This demonstrated the application of a proposed tomographic device for quality assurance in batch crystallisation process. The process of dynamic information could be extracted both from measured USCT data and images providing key insight on process efficiency and process safety.

This study presented a feasibility investigation of ultrasound tomography’s functionality in batch crystallisers. Results are promising for the on-line monitoring and the characterisation of the forming of the suspensions by sound propagation principles. Injection rate and stirring speeds were used as control parameters. The aim was to identify the capabilities of travel-time USCT in the gradually increasing suspension concentration. Results from the experiments were satisfying, showing good potential in distinguishing between phases of different concentrations of particles in suspension. Important conclusions of this study are the determination of:

- Reaction progress by the mean TOF delays values.
- Homogeneity of the medium during injection process by the reconstructions.
- Unwanted by-product formation by the reconstructions.

Even though such a device cannot measure the particle’s size directly, it seems that it could be of great aid in crystallisation processes. It can be used as monitoring of homogeneity or detecting malfunctions and faults. It could work as a quality assurance figure to protect from process malfunctions that could lead to a nonuniform crystal yield. By on-line monitoring, unexpectedly higher formed suspensions can be detected, and, with that, one can control the stirring and injection factors, which are significant adjustable parameters of the process.

On-going research will focus on investigating the effect of a full-waveform ultrasound tomography instrument, utilising attenuation, and back-scattering information. Moreover, higher excitation frequencies will be tested to investigate the penetration depth of the ultrasounds in the process, as it is a crucial adjustment for wave propagation and interaction with particles and slurries.

## Data Availability

Not applicable.
